# A qualitative study on people with opioid use disorders’ perspectives on smoking and smoking cessation interventions

**DOI:** 10.3389/fpsyt.2023.1185338

**Published:** 2023-08-10

**Authors:** Karl Trygve Druckrey-Fiskaaen, Einar Furulund, Tesfaye Madebo, Siv-Elin Leirvåg Carlsen, Lars T. Fadnes, Torgeir Gilje Lid, for ATLAS4LAR Study Group, Vibeke Bråthen Buljovcic

**Affiliations:** ^1^Bergen Addiction Research, Department of Addiction Medicine, Haukeland University Hospital, Bergen, Norway; ^2^Department of Global Public Health and Primary Care, University of Bergen, Bergen, Norway; ^3^Centre for Alcohol and Drug Research, Stavanger University Hospital, Stavanger, Norway; ^4^Department of Respiratory Medicine, Stavanger University Hospital, Stavanger, Norway

**Keywords:** smoking cessation, opioid agonist treatment, qualitative study, systematic text condensation, COM-B, tobacco

## Abstract

**Introduction:**

Smoking-related diseases are major contributors to disability and shorter life expectancy among opioid-dependent patients. Smoking prevalence is considerably higher for opioid-dependent persons than among the general population, and only a minority quit smoking in treatment settings. Studies show that pharmacological smoking cessation interventions have modest success rates. This study aimed to investigate patients’ receiving opioid agonist therapy perspectives on factors affecting behavior and decisions related to smoking cessation, and their experiences with smoking cessation.

**Methods:**

This is a qualitative study using semi-structured individual interviews. The participants were asked, among others, to elaborate on the participants’ thoughts about smoking, previous attempts to quit tobacco use, and what could prompt a smoking cessation attempt. We analyzed the transcripts with systematic text condensation. The Standards for Reporting Qualitative Research and the Consolidated Criteria for Reporting Qualitative Research guidelines were followed. Opioid-dependent patients receiving opioid agonist therapy in outpatient clinics were invited to participate using a purposive sampling method. In total, fourteen individuals participated in this study.

**Results:**

We identified six themes which were: (1) reflections on how smoking affected decisions, (2) smoking and its impact on physical and mental health, (3) the economy as a motivator to stop smoking, (4) emotions, desires, and habits related to smoking, (5) knowledge of smoking, smoking cessation, and quit attempts, and (6) social factors influencing the participants’ choices and activities. The participants were well informed about the consequences of smoking and had some knowledge and experience in quitting. The participants’ pulmonary health was an important motivational factor for change. Withdrawal symptoms, anxiety, and fear of using other substances discouraged several from attempting to quit smoking. In contrast, social support from partners and access to meaningful activities were considered important factors for success. Few reported being offered help from health professionals to make a smoking cessation attempt.

**Discussion:**

Experiencing social support, being encouraged to quit smoking, and patients’ concerns for their physical health were important reasons for wanting to quit smoking. Smoking cessation interventions based on patient preferences and on the behavior change wheel may enable a higher success rate among patients receiving opioid agonist therapy.

## 1. Introduction

Patients with opioid dependence have a markedly lower life expectancy than the general population (1, 2). Opioid agonist therapy (OAT) substantially reduces mortality, but the morbidity and mortality rates remain higher than for the general population (3–5). An estimated 85% of patients receiving OAT smoke tobacco (6). A meta-analysis on asthma and chronic obstructive pulmonary disease (COPD) among illicit opioid users estimated an asthma and COPD prevalence of 20 and 18%, respectively, for persons who inhaled opioids (7). An autopsy study on patients who died while in OAT treatment found that 41% of the patients had emphysema (3). In a Swiss sample of 125 patients undergoing OAT, 30% received a diagnosis of COPD following spirometry, highlighting the significant impact of pulmonary diseases on their overall disease burden (8). Persons receiving OAT are exposed to additional health risks such as hepatitis C infections (9) and mental health disorders (10). Smoking is associated with the development and progression of liver disease (11) and has been shown to negatively affect mental health (10, 12, 13). Thus, reducing the rates of tobacco smoking among patients in OAT provides a potential of reducing several health risks in this population.

Pharmacologic and behavioral interventions effectively increase smoking cessation in the general adult population (14). However, smoking cessation seems harder to achieve among people with opioid use disorders (15, 16) including patients receiving OAT (17, 18). Pharmacotherapies, such as nicotine replacement or varenicline have modest effect rates for smoking cessation among methadone maintained, and other patients with opioid use disorder (16, 17). The pro-smoking social norms, social networks composed of predominantly smokers, psychological distress, perceived stress and intolerance of withdrawal discomfort have been identified as some of the challenges facing opioid-dependent patients wanting to quit smoking (16). In addition, growing evidence indicate that interactions between nicotine and OAT medication increase smoking (16, 19–21); providing a possible explanation for the low quit rates among patients receiving OAT. Further, patients receiving OAT are rarely offered smoking cessation interventions (22, 23). Utilization of smoking cessation services among patients receiving OAT is low: among patients in OAT treated with methadone, who were referred to a quit line, about one fifth utilized it (24). Few patients with opioid use disorder treated with buprenorphine, used behavioral support, including stop-smoking programs and counseling (25). Experiences with smoking cessation among persons with other substance use disorders (SUD) may provide additional insights to consider when designing specific interventions for persons with opioid use disorders.

The research on smoking cessation among patients with SUD often pool results from different treatment modalities (inpatient, outpatient, community, OAT) and use of other substances such as alcohol, cannabis, stimulants and opioids, making it difficult to identify specific interventions for specific patient groups (14, 26, 27). Among patients with SUD 50% were seriously considering quitting smoking (28) and 79% desired to quit (29). Almost one-half had attempted to quit during the past year (30). A systematic review of qualitative studies indicated that patients with SUD are motivated to quit smoking but often experience a lack of support from health professionals or experience discouragement (27). In addition, the patients differed in their views of the timing of smoking cessation with other SUD treatments (27, 31). In some studies, patients preferred concurrent smoking cessation interventions and treatment of other SUD, whereas others felt smoking cessation interventions should be delivered after treatment for other SUDs (27, 31). Among former smokers, concerns for physical health, experiencing the addictiveness of nicotine, and a desire to improve physical fitness were some of the reasons for quitting (32). Conversely, not experiencing negative health consequences appears to be an incentive to maintain smoking habits – with smokers screened for pulmonary cancer without signs of cancer often interpreting negative results as indicating that smoking was less harmful to them (33). Similarly, in a diagnostic study among a Swiss cohort of people receiving OAT, the participants were asked about readiness for health behavior changes in case of a chronic pulmonary obstructive disease (COPD) diagnosis before spirometry. Only a minority of the patients expressed interest in smoking cessation, but the majority were interested in COPD self-management courses, pharmacological COPD symptom treatment, and lifestyle changes (6). Among smokers in general, behavioral interventions, when used as an adjunct to pharmacotherapy, appear to show promising results in improving smoking cessation rates (34). Additionally, empowering patients with opportunities to adopt healthy behaviors has been found to aid smoking cessation. However, the success of these behaviors largely depends on the individual’s motivation and capability within their specific context (35). To better tailor specific interventions for smoking cessation aimed at patients receiving OAT, there is a need more specific information about their thoughts and beliefs about smoking and experience with smoking cessation.

Given the multitude of factors impacting smoking cessation among patients receiving OAT, a standard taxonomy of behavioral change techniques helps define and design smoking cessation interventions (36, 37). The Behavior Change Wheel framework, including the capability, motivation, and opportunity model (COM-B), has been proposed as a theoretical framework to characterize and design behavior change interventions (38). The wheel’s hub describes factors influencing behavior that could provide targets for interventions; the next layer of the Behavior Change Wheel comprises intervention functions, whereas the outer layer identifies different policies that one can use to deliver these intervention functions (39). COM-B is frequently used to map and identify facilitators and barriers to behavioral change from the practitioner’s perspective (40, 41). However, there is also increasing use of the model to map patients’ perspectives (42, 43). The framework has also been used in studies on smoking cessation among patients with alcohol and illicit drug use (44) and in a review of smoking cessation interventions (35).

This study aimed to investigate the prerequisites for health behavior changes of patients receiving OAT. More specifically, factors affecting behavior and decisions related to smoking cessation, and their experiences with smoking cessation. There is a lack of knowledge about barriers and facilitators for smoking cessation experienced by patients who receive OAT.

## 2. Materials and methods

### 2.1. Design and setting

This is a qualitative study using semi-structured individual interviews. This study is a part of the ATLAS4LAR project aiming to improve health among people with opioid use disorder receiving OAT (45). The authors, patient representatives recruited from patients receiving OAT, and research nurses developed the semi-structured interview guide (Supplementary file 1) in collaboration, which focused on the participants’ perspectives on exercise, nutrition and smoking, and their motivation for changing these habits. This paper presents the results related to smoking and smoking cessation.

The ATLAS4LAR project recruits patients from OAT outpatient clinics in the Norwegian cities of Bergen and Stavanger to a prospective cohort and OAT health registry. Patients are included in the cohort and health registry when they have given written consent and have completed an initial health assessment. The health assessments are repeated yearly. The participants of this study were recruited from this cohort. The OAT outpatient clinics are located in the districts of the cities to provide integrated care and treatment for opioid dependence including dispensation of methadone, buprenorphine, and long-acting morphine, close to where the patients live. Patients usually receive their OAT medication under the supervision of nurses and social workers at the OAT clinics. In addition, the clinics are staffed with consultants specialized in addiction medicine and junior physicians training in addiction medicine. Some clinics are staffed with psychologists as well. Due to COVID-19 restrictions, patients at times received their OAT medication at home delivered by nurses ambulating from the clinics.

### 2.2. Study sample

We aimed to include a purposive sample of OAT clients in Bergen and Stavanger, reflecting the age and gender distribution of the clinics (mean age of 47 years and one third females). In addition, we aimed to recruit patients with and without other substance use disorders, patients motivated for lifestyle changes and those who were not. All patients at the OAT clinics are offered yearly health assessments conducted by the research nurses, who work partly as clinicians and partly as researchers. Patients were eligible to participate if they had completed at least one health assessment, were interested in sharing their thoughts on lifestyle changes and could complete an hour-long interview. There were no specific exclusion criteria. Due to COVID-19 restrictions, the research nurses contacted possible participants by telephone, informed them about the study, and invited them to participate. Interviews were conducted in person, but with COVID-19 measures in place, such as symptom screening prior to the interview, increased distance between persons, and wearing a face mask. The Regional Ethical Committee (REK sør-øst #155386) granted ethical approval for this project. Participants signed an informed consent form prior to participation.

### 2.3. Data collection

In January and February 2021, three research nurses with training in qualitative interviewing conducted individual interviews at the OAT outpatient clinics, and audio-recorded these interviews. The research nurses were all females and known to the patients from health assessments completed prior to inclusion in this study. The participants were informed that the interview included the broader topic health behavior and the three sub-topics smoking, physical activity, and nutrition. The topics of the interview-guide related to smoking were the participants’ thoughts about smoking, previous attempts to reduce or quit tobacco use, whether smoking cessation aids had been offered by healthcare workers, intentions to quit smoking, what could prompt a smoking cessation attempt, and smoking of substances other than tobacco. Interviewers were instructed to attempt to explore all topics. They were allowed to provide prompts if necessary to help the participant. Fourteen interviews were conducted. A 37 year-old male participant ended the interview after 12 min, after completing half the smoking questions. As the participant did not withdraw consent, his responses were included in the analysis. Thirteen participants completed the full interview, with a mean interview duration of 37 min.

### 2.4. Data analysis

**Figure 1:**
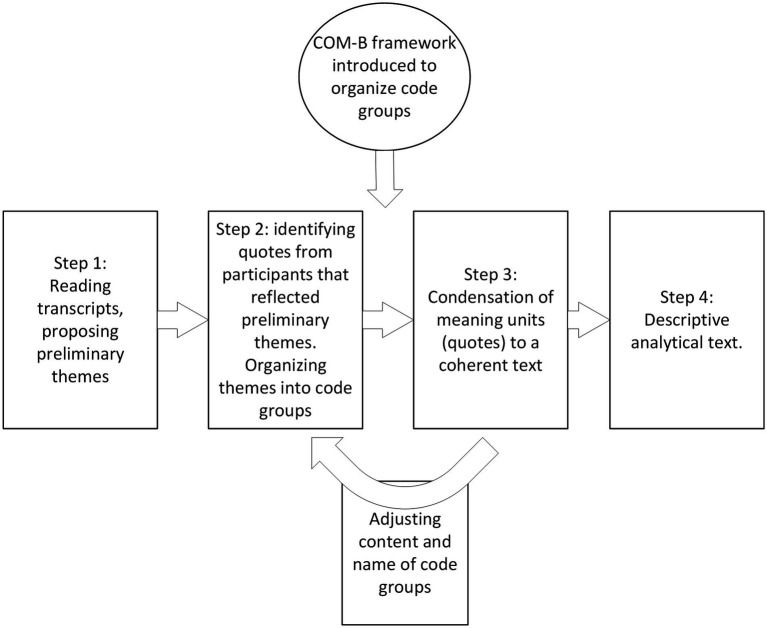
Schematic overview of the analytical process using systematic text condensation.

**Figure 2:**
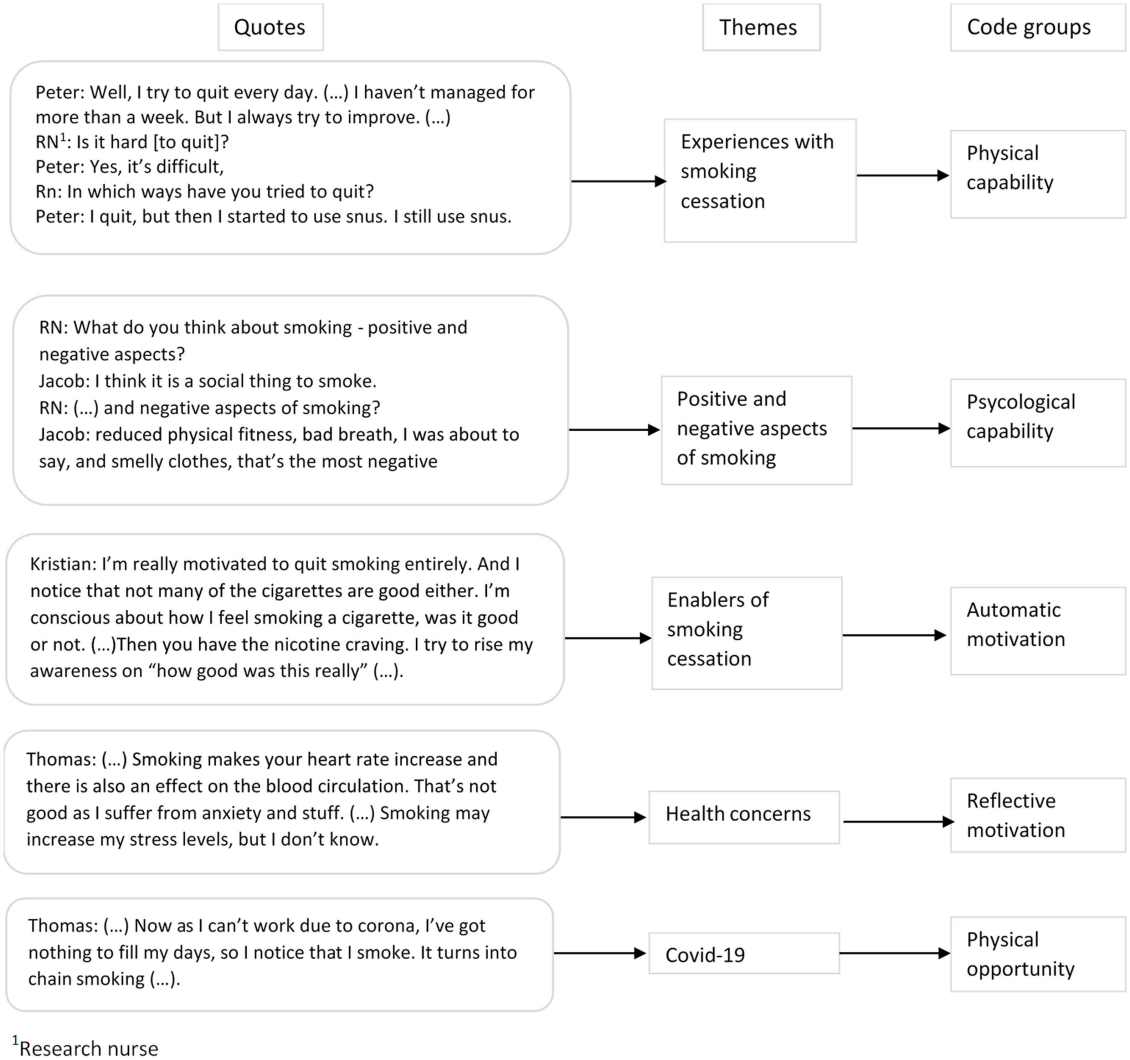
An illustration of how themes and codes were developed during the analytical process.

Recordings were labeled with a pseudonym reflecting the participants’ gender and transcribed verbatim by four of the authors (EF, S-ELC, REN, and KD-F). We used NVivo software versions 12 and 20 (RRID: SCR_014802) when working with the transcripts to facilitate a collaborative analytical process. Due to COVID-19 restrictions and geographical distance between the authors, they mainly met via video conferences. We applied systematic text condensation in the analysis. This is a systematic, step by step approach suitable for thematic cross-case analyses (46, 47). The analysis consisted of four main steps (Figure 1). First, the authors read the transcripts to familiarize themselves with the material. Based on this reading, the authors presented and discussed the preliminary themes they had identified in the transcript and, through dialog, agreed on six themes for further analysis. Second, the authors re-read the transcripts to identify meaning units, which were coded to the preliminary themes. While extracting meaning units and elaborating the preliminary themes into code groups, we noted that they fit well within the COM-B framework (38). During the third step, condensation, the first author used the meaning units to create condensates containing the nuances of the code groups and subgroups. The other authors were repeatedly consulted during this process to discuss the coding groups’ names and delineation. Finally, the condensates were used to create a descriptive text to elucidate the study questions. An illustration of how the themes and codes were developed is shown in Figure 2. The Consolidated Criteria for Reporting Qualitative Research (COREQ) were used in writing this manuscript (48). The COREQ checklist is presented in Supplementary file 2.

## 3. Results

At the time of the study all participants met the criteria for opioid use disorder according to the international classification of diseases, 10th edition. All participants received OAT. The median age of the participants was 49 years (range 30–60). Three of the participants were females. Most participants (11 out of 14) had completed 10 years of education or more. All reported stable housing conditions.

Most participants (13 out of 14) reported smoking tobacco at least three times a week. A quarter reported using cannabis more than three times a week. One-half of the participants reported not using alcohol, cannabis, stimulants, or benzodiazepines during the past 30 days. The majority (13 out of 14) of the informants did not use opioids other than OAT medication.

Participants’ characteristics are shown in Table 1.

**Table 1:** Characteristics of the participants (*n* = 14).

Age, median (range)	49 (30–60)
Females	3 of 14
Mean duration interview (minutes)^a^	39
OAT medication	
Methadone	4 of 14
Buprenorphine	10 of 14
Education	
Not completed basic education^b^	3 of 14
Completed basic education^a^	5 of 14
High school^c^	4 of 14
University	2 of 14
Debt difficulties^d^	4 of 14
Living alone	6 of 14
Debut age, median (range)^e^	
Opioids	25 (14–32)
Cannabis	14 (12–30)
Tobacco	13 (10–27)

a Mean time for the 13 participants who completed the interviews. One participant withdrew after 12 min.

b In Norway, the first ten school years are mandatory for all pupils.

c Grades 11–13.

d Not able to pay off legal or illegal debt.

e The age at which the patient started using the substance.

The following themes were identified: reflections on smoking and how this affected decisions, smoking and health impact, and the economy as a motivation for quitting smoking, which aligned to the reflective motivation construct. Emotions, desires, and smoking habits fit the description of the automatic motivation construct. Furthermore, the themes knowledge about smoking, smoking cessation, and quit attempts aligned with the capabilities construct. Finally, social factors influencing the participants’ choices and activities aligned to the opportunities construct of the behavior change wheel.

### 3.1. Reflections on how smoking affected decisions, reflective motivation

Several participants talked about feeling good when they smoked and how they enjoyed the ritual of rolling a cigarette, sitting down, and smoking it. Jacob described why he liked smoking:

*“… I enjoy smoking; it’s that simple. I like taking a cigarette. Just like others drink a beer, I, instead, smoke a cigarette.”*

Many strongly desired to quit smoking but noted that it was tough. When asked what they thought would be helpful when quitting smoking, most responded that regular daily routines and activities were necessary. Regular activities could contribute to reducing smoking on their own, and too much spare time, in their experience, leads to increased smoking. Among those who wanted to quit smoking, it was essential to avoid smoking long enough to see the benefits and convince oneself that they could manage their lives without cigarettes. One participant sums up his thoughts about smoking:

*“Smoking is bad… It will not make you any happier. Nevertheless, I will smoke when I get outside after this interview. However, I will start to ponder what I get from smoking – well – another addiction.” (Anne).*

#### 3.1.1. Smoking and its impact on health

Most participants experienced shortness of breath and poor physical fitness and related this to smoking. They described how a slight increase in physical activity caused wheezing and chest pain. Several participants described heavy coughs causing retching in the morning, and only after two or three cigarettes did the coughing abate. A patient had a family history of cancer and cardiopulmonary disease and was worried about his own risk. Others were worried that smoking would negatively affect lung function. A couple of patients had managed to reduce the number of cigarettes smoked and aimed to stop smoking within the following months. A participant reflected on smoking and its health impact as follows:

*“Just after two puffs I start coughing, coughing, and coughing. Why does one smoke at all? Suffocating oneself. Well, I try to smoke as little as possible.” (John).*

Although the damaging physical effects were the most predominant concern among the participants, some also reflected on how smoking affected stress and anxiety. They described how smoking increased heart rate and affected anxiousness and bodily tensions, concluding that smoking may lead to more stress. At the same time, a participant responded as follows when asked why he would not make a quit attempt:

*“… because I’m so dependent, that I have no desire to [quit] due to stress and withdrawal symptoms.” (Mark).*

Furthermore, some were not currently worried about their health. In contrast, they interpreted normal findings in lung function tests as an indication that smoking did not harm them personally. When asked what could make him accept an offer to stop smoking, Jacob responded:

*“[I would stop] if I had gotten troubles with my lungs or something similar.”*

#### 3.1.2. The economy as a motivator to stop smoking

Most participants relied on social benefits. They knew that cigarettes and tobacco are considerable expenses, and that smoking cessation would free up several thousand Norwegian kroner monthly. Several patients believed that saving money would be a motivation to quit smoking, and a few reported the high cost of smoking as a motivating factor to stop smoking. They were optimistic about accepting an offer of help with smoking cessation, as it would save them much money. Some reported that they had not tried nicotine replacement products for smoking cessation due to the cost of these products, but if they received these for free, they would be interested in making a quit attempt. Peter explained:

*“Yes, they [smoking cessation medications] are so expensive, you know… So, if a [nicotine] patch would cost as much as snuff, I would have chosen the patch.”*

### 3.2. Emotions, desires, and habits related to smoking, automatic motivation

For some, smoking had become an automated habit, and many noted that it made their days easier to cope with. Erik had not reflected on why he was smoking:

*“That is a good question. I have been addicted [to cigarettes] for forty years. That is a long time. So why? I actually cannot answer that question.”*

Automated habits appeared to play an essential role in sustaining smoking: combining cigarettes and coffee in the morning was pleasant for many. They described that some cigarettes tasted so pleasantly that they felt the urge to take another puff and then another until they were addicted to the puffing. Changes in smoking habits were often subtle. A participant described only intending to smoke outside. However, after a few months, he smoked and drank coffee while sitting in front of the television. Others described how they woke up due to nocturia and then had to smoke a cigarette to feel sleepy again. They described falling asleep again faster after smoking, although at the same time, noticing that smoking did not do them well. Some also reported smoking cannabis and tobacco in the evening to make them relax and fall asleep more easily. A participant summarized the emotions smoking evoked:

*“In the morning, together with coffee, the cigarettes are delicious. Nevertheless, I have to tell you; I smoke eight cigarettes a day. Some of those taste awful, and I get a bad feeling when I smoke.” (Steve).*

### 3.3. Knowledge of smoking, smoking cessation, and quit attempts, physical and psychological capabilities

Participants had tried different smoking cessation products, such as chewing gum and nicotine patches. In some cases vapers and e-cigarettes were used by the participants as an alternative to traditional smoking. A couple of participants had tried varenicline tablets. Oliver talked about his difficulties using chewing gum:

*“Well, it does not work for me, because I have no teeth.”*

Several reported unpleasant taste and lack of effect from nicotine replacement product, in particular chewing gum:

*Thomas: «I’ ve tried nicotine chewing gum, it was like chewing on the cigarette filter. It was no good.”*

*John described: «…it tasted so awfully and did not reduce my cigarette craving, so I gave it up pretty quick.”*

Many were able to reduce the number of cigarettes smoked when they used smoking cessation products but noted that it was difficult to quit entirely. A participant shared how she changed habits to reduce smoking:

*“Well, I buy a pack of twenty cigarettes, and then I take five and five. On several days, I notice when going to bed that I still have one [cigarette] left. Then, it is a little like, “Oh, I only smoked four,” so yes, at the moment, I am happy with it.” (Christine).*

When asked about their knowledge about smoking, many conveyed that smoking was dangerous, causing shortness of breath, cough, bad breath, reduced fitness, reduced taste, and smelling clothes. The experience of the addictiveness of smoking made some afraid that quitting would make them start using other substances. Despite this, they had attempted to quit repeatedly:

*“However, just quitting smoking, is quite hard. I am afraid I would resort to other things, such as other drugs, to stimulate the cravings. Now I do it like this; I only bring a few cigarettes when I leave home. That way I will get through the day, but I smoke anyhow.” (John).*

### 3.4. Social factors influencing the participants’ choices and activities, social, and physical opportunities

A few participants were offered nicotine patches during hospital admissions, as hospitals have a non-smoking policy. However, most patients responded that smoking cessation never was a topic during consultations with primary care doctors or at OAT clinics. Only one participant reported asking a doctor for help with smoking cessation. Respondents believed they were not offered help with smoking cessation because the doctors assumed they already had enough to deal with. When asked about her thoughts on why no one had asked about smoking cessation, Anne replied:

*“Well, I guess it is because there are so many problems in my life, and tobacco does not kill me. So, when none of the other things kill me, this will not either.”*

Several participants reported that partners, roommates, and health workers influenced their smoking habits. One participant put it this way:

*“I will have to get better – to find the opportunity to quit smoking – because I know that my partner wants us both to reduce smoking. In addition, she has already done it. Therefore, if I am not doing it for my own sake, I will try it for our sake.” (Thomas).*

At the same time, others responded that because they had no partner, there was little motivation to attempt quitting:

*“… if I had a partner, who did not smoke – I would have quit, right away. I she had told me that she’d leave me if I did not quit (laughing). … I have always wanted to quit smoking, but I’ve only had myself to care about. I’m sloppy taking care of my body when it comes to drugs and all that” (Steve).*

A participant reflected on her pregnancy experience when she increased smoking as health workers reminded her to reduce smoking:

*“But I know it causes damage: each puff you take narrows the umbilical cord, stopping the baby’s oxygen supply. But I do not know, what it takes to scare you into stopping smoking, because smoking is no good” (Anne).*

Robert reflected on how a visit with the research nurse impacted his smoking:

*“After the last visit I managed to wait two days, before I had my next cigarette.”*

Loneliness was a strong motivator for smoking. Two participants said they smoked more frequently due to COVID-19 pandemic measures as activities were closed. They tended to chain-smoke, using smoking as a substitute for social interaction.

*Thomas: «… it depends how you spend your days. If you have much spare time, no job, and few activities, you very soon start smoking something.”*

Jeanette also noted that the usual cannabis smoking in the evening extended into the day:

*“It is an evening thing [smoking cannabis mixed with tobacco]. If it turns into more than an evening thing, my days are gone. I’ve been smoking much during the whole day, now with Corona, to have something to do.”*

Participants reflected on how living conditions affected smoking habits depending on where they lived and with whom:

*“When I’m at me ex-boyfriends place I barely smoke because I feel good without stress. But if I have to stay in the shelter, I might smoke 20 cigarettes a day.” (Anne).*

*“Yes, well I smoke too much now. I’ve got someone living at my place, who smokes continuously [tobacco and cannabis] from he gets up in the morning until he goes to bed.” (Harald).*

## 4. Discussion

This study investigated the prerequisites for health behavior changes, specifically patients’ experiences and factors affecting behavior and decisions related to smoking cessation among patients receiving OAT. We found that patients’ concerns for physical health and experiencing social support were important reasons for wanting to quit smoking. The high cost of cigarettes was a motivating factor for quitting, but the cost of nicotine replacement products was a barrier. Smoking was also a coping strategy for stress and withdrawal symptoms from other substances. Other barriers were lack of support from health care providers, loneliness, and living conditions. Many patients receiving OAT held positive attitudes toward smoking cessation and had some knowledge of how to reduce smoking.

Although persons with SUDs are perceived as a “hard-to-reach” population regarding smoking cessation (15, 16, 26, 49, 50), our study indicates that patients receiving OAT have motivations, capabilities, and opportunities to make smoking cessation attempts. Among patients receiving buprenorphine, motivational interviewing and cognitive behavioral therapy increased motivation to make a cessation attempt (51). When behavioral therapy was given in addition to nicotine replacement therapy it did not change the odds of smoking cessation compared to nicotine replacement therapy alone among patients receiving methadone (17). Pharmacotherapy with nicotine replacement products and varenicline increased the odds of smoking cessation for patients with opioid use disorders and patients receiving OAT, however adherence to cessation treatment was a major mediator of cessation success (16, 17). The participants in our study presented missing teeth and bade taste of nicotine replacement as factors affecting adherence to cessation treatment. The factors influencing their decisions on smoking cessation are similar to those of other smokers (33, 52–54). Among those factors, self-experienced smoking-related health concerns were a motivator for change among participants of our study. However, knowledge of the risks of smoking did not influence cessation behavior in the absence of self-experienced health symptoms. Such behavior is common among smokers (53, 54). At the same time, smoking to relieve stress and difficulties controlling other addictions is a common reason for not attempting to quit (55–57), as also observed in our study sample. This points to the significance of smoking as a coping strategy (58), and the importance of focusing on how patients can develop alternative strategies (59).

Healthcare providers often convey low expectations regarding SUD patients’ ability and motivation to quit smoking (60, 61). Thereby, smoking cessation is not seen as part of the treatment culture (60, 61). There was a perception among health care providers that smoking cessation is less important compared to treatment of the primary addiction, and that clients do not prioritize smoking cessation (29, 61). Our participants reported little support from healthcare providers on smoking cessation. This is a lost opportunity. First, quitting smoking as part of the personal treatment plan is associated with higher smoking cessation rates (53). Second, there is growing evidence for the usefulness of concurrent smoking cessation interventions during treatment for other addictions (26, 54, 62, 63): tobacco cessation interventions do not appear to influence the abstinence from alcohol and other drugs (26, 62). At a cellular level, the bidirectional interactions between the nicotine and opioids systems provide another argument for concurrent treatment of nicotine and opioid dependence. These cellular interactions explain in part the behavioral and physiological effects of dual use of nicotine and opioids (64). Patients in residential treatment considered being in treatment a good opportunity to attempt smoking cessation, as part of a healthier lifestyle (54). A smoking cessation intervention appeared to increase the number of drug-free days among stimulant users (63). Anne’s description of how she increased smoking during pregnancy is an example of how non-motivational approaches to smoking cessation increase a patient’s resistance to change (65), and how a patient’s resistance may increase a health professional’s confrontational behavior (66).

Several participants identified social support and having a partner as important reasons for making a cessation attempt. Being married was positively associated with smoking cessation (67). However, smoking is more socially accepted in SUD treatment facilities, and its social acceptance within the group may constitute a barrier to smoking cessation (56, 68). Implementing “smoke-free” grounds could be an effective measure, as residential treatment facilities with policies restricting smoking on the premises observed reduced smoking rates among clients (69, 70). At the same time, treatment for SUD was associated with lower rates of smoking cessation compared to those with SUD not receiving treatment (67). The authors postulated the use of tobacco to alleviate withdrawal symptoms from other substances and the lack of professional support as explanations for this difference (67). A Cochrane review concluded to the contrary that smoking cessation interventions provided to people in treatment or recovery for drug or alcohol dependencies would reduce the health consequences of smoking. Providing smoking cessation did not affect the abstinence rates from other drugs and alcohol (62).

Our study indicates that patients know about smoking cessation interventions, including medication and behavioral techniques such as tapering the daily number of cigarettes. Several studies have identified an association between the level of education and smoking cessation (67, 71). Patient education may assist in smoking cessation (68), thus indicating that patient education should be part of smoking cessation interventions in addition to motivational interviewing, behavioral therapy (51), and interventions to increase adherence with the cessation program (17). However, given healthcare providers’ crucial role in supporting patients’ smoking cessation attempts and the fact that staff may lack the preparedness, knowledge, skills, and attitudes to provide smoking cessation interventions, it appears that education and training of healthcare providers is essential to increase the success of smoking cessation programs (29, 60, 61). Our findings on barriers and enablers for smoking cessation among Norwegian patients receiving OAT are consistent with other studies from the United States (68), England (29), and Australia (57), although the participants of these studies are not all OAT recipients. Our study increases the knowledge on barriers and facilitators of smoking and smoking cessation among patients who receive OAT. By mapping the results using the COM-B nomenclature (39), we identified several components that could be targeted through specific smoking cessation interventions for patients receiving OAT. Firstly to increase the accessibility to smoking cessation by making it part of the standard care at OAT clinics and providing smoking cessation products for free, thus improving the patients’ opportunities to quit. The patients’ motivation and capability could be enhanced by offering regular appointments with health care professionals at the OAT clinics to support the cessation attempt. Educating staff at OAT clinics on how to provide smoking cessation therapy and the challenges patients receiving OAT face when they attempt to quit smoking could improve the capability and motivation of the staff, and hence the opportunities for the patients. Declaring the premises of the OAT clinics as smoke-free and encouraging smoke-free social activities such as physical activity or cultural activities are other interventions aimed at the patients’ opportunities and motivation.

This study has strengths and limitations. Research nurses recruited the participants by direct contact at OAT outpatient clinics by purposive sampling to overcome some of the recruitment challenges associated with inviting hard-to-reach populations. Purposive sampling uses specific criteria to select eligible participants. We aimed to reflect the general OAT population in age and gender distribution. Finally, we wanted to recruit patients motivated for lifestyle changes and those with no such interest as well as patients with comorbid substance use and those without. The sample size was considered using the concept of information power (72). It could also be argued that higher number of participants could provide more details. The sample may not precisely reflect the general OAT population, and the results of this study will apply to patients similar to the participants. At the same time, our sample is comparable to the Norwegian OAT population: the mean age of the Norwegian OAT population is 47 years, approximately one-third are females, and 80% of OAT patients nationally report stable housing conditions (73).

Social desirability (74) could affect the respondents’ answers. Here, the familiarity of the research nurses might allow them to relax and talk without consideration of the expected answers. However, being familiar with the research nurses could also lead to answers that appear more socially desirable. Given the research project’s focus on lifestyle changes and the researchers’ interest in smoking cessation, there is a potential to specifically search for information on these topics, missing other aspects of importance to the participants.

The COM-B framework (38) has potentially influenced the reporting of the results, as codes were organized according to the COM-B framework. It is, however, essential to note that the interview guide was developed without the COM-B framework and that this framework was first used following the completion of the second step of systematic text condensation (46), in which the authors had identified meaning units and coded them to the preliminary themes.

## 5. Conclusion

An estimated 85% of patients who receive OAT smoke tobacco. Simultaneously they have among the lowest quit rates. Standard smoking cessation treatments, such as pharmacological interventions, have modest success rates, and behavioral interventions alone do not increase the odds of smoking cessation. Studies using multiple combined interventions specifically aimed at patients at OAT clinics are lacking. A possible way of increasing the cessation rates could be to design specific interventions for patients in OAT, integrate the smoking cessation interventions in the OAT clinics, consider the multiple barriers and facilitators for smoking cessation, mapped using COM-B, and test such interventions in randomized controlled studies. In addition, strategic initiatives are imperative to guarantee access to resources that foster smoking cessation among vulnerable groups. Implementation of effective policies, including the provision of public funding for nicotine replacement products, is a key strategy in addressing the unique needs of these populations. By employing such comprehensive strategies, we can significantly enhance the likelihood of successful smoking cessation among vulnerable groups.

## Data availability statement

The raw data supporting the conclusions of this article will be made available by the authors, without undue reservation.

## Ethics statement

The studies involving human participants were reviewed and approved by Regional ethical committee (REK-sør øst), approval #155386. The patients/participants provided their written informed consent to participate in this study.

## Author contributions

KD-F, EF, TM, S-EC, LF, and TL were involved in the study’s design and data analysis and contributed to the manuscript. KD-F wrote the first draft and led the writing process. All authors contributed to the article and approved the submitted version.

## Funding

This study was funded by the Regional Health authorities of Western Norway.

## Conflict of interest

The authors declare that the research was conducted in the absence of any commercial or financial relationships that could be construed as a potential conflict of interest.

## Publisher’s note

All claims expressed in this article are solely those of the authors and do not necessarily represent those of their affiliated organizations, or those of the publisher, the editors and the reviewers. Any product that may be evaluated in this article, or claim that may be made by its manufacturer, is not guaranteed or endorsed by the publisher.

## Abbreviations

OAT: Opioid agonist therapy
SUD: Substance use disorder
COPD: Chronic obstructive pulmonary disease
COM-B: Capability opportunity motivation for behavior framework
COREQ: The consolidated criteria for reporting qualitative research
