# Supplementary material for: A qualitative study on people with opioid use disorders’ perspectives on smoking and smoking cessation interventions
Source: Front Psychiatry. 2023 Aug 10;14:1185338. doi: 10.3389/fpsyt.2023.1185338 (PMC10447904; doi:10.3389/fpsyt.2023.1185338)
Supplement: Supplementary file 1 [file Data_Sheet_1.docx]

**Supplementary file 1: English translation of the interview guide, originally written in Norwegian.**

**Interview guide**

(Please note that everything in *italics* is only for information and help, but is not mandatory in the interview)

**Patient version**

Interviewer says: Today's date is………..

Are you ready to begin?

Topic 1: Health and lifestyle

What do you think about your health today? *(Try to find out what the individual thinks is good health)*

What do you believe affects your health? (Positive and negative aspects)?

If you were to make changes to improve your fitness and health, what would you do?

You have now given us some general information about your health. For the project we are working on, we would like to know more about some topics that we would now like to ask you about:

Topic 2: Physical activity

Can you briefly describe previous experiences, both positive and negative, that you have had with physical activity and training? *(the important thing here is to get hold of experiences with activity, and whether the experiences were positive or negative, but not more "peripheral" stories related to the activity)*

*Try to get information on*:

- *Type of training, intensity, and frequency*

*- Where and under what circumstances did the participant do physical activity?*

How would you describe your current physical activity and exercise practices?

• Do you walk much in everyday life?

• Do you exercise regularly (organized or alone)?

• What activities/types of exercise do you like?

• What keeps you active?

• What prevents you from being active?

• Do you have a goal to become more active in your everyday life?

What does it take for you to get more active in your everyday life?

Do you have any input on how we can facilitate increased physical activity in people receiving OAT treatment?

*• At the clinic with health worker? Group offer - individually, divided by gender, what time should such training be carried out? What activities?*

*• Are you familiar with the offers available today? Which ones have you used?*

What could prevent you from taking part in an exercise program at the OAT clinic?

Topic 3: Smoking

What thoughts do you have about smoking *(positive and negative)*?

Do you smoke anything other than tobacco, how often?

Have you tried to reduce your use of tobacco or quit smoking?

*• If yes:*

- Could you tell how it went and what experiences you had?
- Did you use any medication to stop smoking?

*• If no:*

- Could you say something about why you haven't tried to stop smoking or cutting back?

If you smoke something other than tobacco: What will it take to reduce or stop using it?

Have health personnel (such as a GP, therapist at the OAT clinic, etc.) talked to you about smoking/smoking cessation?

Have you been offered help to stop smoking?

*• If yes:*

- Could you say a little about how you experienced this?

*• If no:*

- What would you think if you were asked? Could you say a little about what you think is the reason why no one has asked?

What will it take for you to stop smoking or cut back?

Would you consider making an attempt to reduce or quit smoking in the next year, if you were offered something that could help you with this?

| *YES* | *NO* |
| --- | --- |
| - What help will you need to stop smoking? (*examples can be medication, counselling, group therapy, information material, smoking cessation hotline)* - What do you think about using medication to stop smoking? *Examples of medications are nicotine replacement (patch, chewing gum, lozenge, oral spray, inhaler) or Varenicline tablets.* *These can be combined in different ways* - Have you tried any medications before that would not be relevant now? - We are considering offering (free) nicotine replacement or Champix to those who want to try to quit smoking. What do you think would be the best way to do this? *(possible alternatives would be to pick it up at a pharmacy yourself or have it handed out at the OAT clinic when OAT medication is picked up. You can get it for a few days at a time or for several weeks at a time)* | • Why not?  • What does smoking give you?  • What are the positive aspects of smoking?  • What are the negative aspects of smoking?  • Could reducing/quitting smoking become an alternative for you at a later time? |

Topic 4: Diet

What do you think about your diet today?

Can you tell us a bit about what you usually eat and drink?

• How many meals a day?

• Do you often cook yourself? *If no:* why not? Do you have access to a kitchen?

• What kind of food do you cook?

What opportunities do you have to prioritize your diet?

• How do your finances affect what food you buy?

• Do you have ailments that make it difficult to eat? *(examples could be dental health, stomach problems from certain foods, swallowing difficulties, eating disorder, something else)*

If you were to change something about your diet, what would you change?

We want to start a project to help the OAT patients to have a healthier diet. What do you think such a project should offer?

Closing question

Is there anything else you'd like to add to what we've talked about today?
