# Supplementary material for: A qualitative study on people with opioid use disorders’ perspectives on smoking and smoking cessation interventions
Source: Front Psychiatry. 2023 Aug 10;14:1185338. doi: 10.3389/fpsyt.2023.1185338 (PMC10447904; doi:10.3389/fpsyt.2023.1185338)
Supplement: Supplementary file 2 [file Data_Sheet_2.docx]

**Supplementary file 2**

**Consolidated criteria for reporting qualitative research (COREQ)(1):**

| No. | Item | Guide questions/ descriptions | Responses |
| --- | --- | --- | --- |
| **Domain 1: Research team and reflexivity** | | | |
| 1. | Interviewer/facilitator | Which author/s conducted the interview or focus group? | None, research nurses affiliated with the research group conducted the interviews. |
| 2. | Credentials | What were the researcher’s credentials? *E.g. PhD, MD* | KLTD: MD and Phd-candidate, EF: M.Sc. and Phd-candidate, TM: MD Phd, SELC: M.sc. PhD, LTF: MD PhD, TGL: MD PhD |
| 3. | Occupation | What was their occupation at the time of the study? | All researcher were employed by the Bergen addiction research group. |
| 4. | Gender | Was the researcher male or female? | Both |
| 5. | Experience and training | What experience or training did the researcher have? | The senior researchers (SELC; LTF AND TGL) have previous experience in qualitative research |
| 6. | Relationship established | Was a relationship established prior to study commencement? | Participants knew research nurses from yearly health assessments |
| 7. | Participant knowledge of the interviewer | What did the participants know about the researcher*? e.g. personal goals, reasons for doing the research* | The participants were informed about the reasons for the project (ATLAS4LAR) |
| 8. | Interviewer characteristics | What characteristics were reported about the interviewer/facilitator? *e.g. Bias, assumptions, reasons and interests in the research topic* | No characteristics were reported |
| **Domain 2: study design** | | | |
| 9. | Methodological orientation and Theory | What methodological orientation was stated to underpin the study? *e.g. grounded theory, discourse analysis, ethnography, phenomenology, content analysis* | We used systematic text condensation (2) to guide the design and analysis of the study. |
| 10. | Sampling | How were participants selected? *e.g. purposive, convenience, consecutive, snowball* | The sampling was purposive. See page 4 for further information. |
| 11. | Method of approach | How were participants approached? *e.g. face-to-face, telephone, mail, email* | Participants were approached by telephone and face to face. |
| 12. | Sample size | How many participants were in the study? | 14 |
| 13. | Non-participation | How many people refused to participate or dropped out? Reasons? | This was not specifically recorded. |
| 14. | Setting of data collection | Where was the data collected? *e.g. home, clinic, workplace* | At the patients’ local OAT- clinic (see table 1) |
| 15. | Presence of non-participants | Was anyone else present besides the participants and researchers? | No |
| 16. | Description of sample | What are the important characteristics of the sample? *e.g. demographic data, date* | See table 1 |
| 17. | Interview guide | Were questions, prompts, guides provided by the authors? Was it pilot tested? | The interview guide was semi structured with open end questions. The interview guide was not piloted, but developed together with user representatives. |
| 18. | Repeat interviews | Were repeat interviews carried out? If yes, how many? | None were repeated |
| 19. | Audio/visual recording | Did the research use audio or visual recording to collect the data? | Interviews were audio-recorded |
| 20. | Field notes | Were field notes made during and/or after the interview or focus group? | No |
| 21. | Duration | What was the duration of the interviews or focus group? | The duration was 13-60 minutes. Mean time was 37 minutes |
| 22. | Data saturation | Was data saturation discussed? | The authors discussed saturation during the analysis process. |
| 23. | Transcripts returned | Were transcripts returned to participants for comment and/or correction? | No |
| **Domain 3: analysis and findings** | | | |
| 24. | Number of data coders | How many data coders coded the data? | All authors were involved in the initial steps of the analysis coding preliminary themes. Once the themes were decided upon KTDF and EF extracted meaning units. |
| 25. | Description of the coding tree | Did authors provide a description of the coding tree? | Figures 1 and 2 |
| 26. | Derivation of themes | Were themes identified in advance or derived from the data? | Themes were derived from the data. |
| 27. | Software | What software, if applicable, was used to manage the data? | The NVivio software was used to manage the data. |
| 28. | Participant checking | Did participants provide feedback on the findings? | No |
| 29. | Quotations presented | Were participant quotations presented to illustrate the themes / findings? Was each quotation identified? *e.g. participant number* | Several quotations are presented in the manuscript. Each participant was given a pseudonym which was used to identify the quotes. |
| 30. | Data and findings consistent | Was there consistency between the data presented and the findings? | There is consistency between presented data and the findings (see pages 5-9) |
| 31. | Clarity of major themes | Were major themes clearly presented in the findings? | The major themes are clearly presented with subheadings (see pages 5-9) |
| 32. | Clarity of minor themes | Is there a description of diverse cases or discussion of minor themes? | Diverse cases and minor themes are discussed in the paper (see pages 5-9) |

1. Tong A, Sainsbury P, Craig J. Consolidated criteria for reporting qualitative research (COREQ): a 32-item checklist for interviews and focus groups. Int J Qual Health Care. 2007;19(6):349-57.

2. Malterud K. Systematic text condensation: a strategy for qualitative analysis. Scand J Public Health. 2012;40(8):795-805.
